# A strategy to improve phasing of whole-genome sequenced individuals through integration of familial information from dense genotype panels

**DOI:** 10.1186/s12711-017-0321-6

**Published:** 2017-05-16

**Authors:** Pierre Faux, Tom Druet

**Affiliations:** Unit of Animal Genomics, GIGA-R and Faculty of Veterinary Medicine, University of Liège, 4000 Liège, Belgium

## Abstract

**Background:**

Haplotype reconstruction (phasing) is an essential step in many applications, including imputation and genomic selection. The best phasing methods rely on both familial and linkage disequilibrium (LD) information. With whole-genome sequence (WGS) data, relatively small samples of reference individuals are generally sequenced due to prohibitive sequencing costs, thus only a limited amount of familial information is available. However, reference individuals have many relatives that have been genotyped (at lower density). The goal of our study was to improve phasing of WGS data by integrating familial information from haplotypes that were obtained from a larger genotyped dataset and to quantify its impact on imputation accuracy.

**Results:**

Aligning a pre-phased WGS panel [~5 million single nucleotide polymorphisms (SNPs)], which is based on LD information only, to a 50k SNP array that is phased with both LD and familial information (called scaffold) resulted in correctly assigning parental origin for 99.62% of the WGS SNPs, their phase being determined unambiguously based on parental genotypes. Without using the 50k haplotypes as scaffold, that value dropped as expected to 50%. Correctly phased segments were on average longer after alignment to the genotype phase while the number of switches decreased slightly. Most of the incorrectly assigned segments, and subsequent switches, were due to singleton errors. Imputation from 50k SNP array to WGS data with improved phasing had a marginal impact on imputation accuracy (measured as *r* ^2^), i.e. on average, 90.47% with traditional techniques versus 90.65% with pre-phasing integrating familial information. Differences were larger for SNPs located in chromosome ends and rare variants. Using a denser WGS panel (~13 millions SNPs) that was obtained with traditional variant filtering rules, we found similar results although performances of both phasing and imputation accuracy were lower.

**Conclusions:**

We present a phasing strategy for WGS data, which indirectly integrates familial information by aligning WGS haplotypes that are pre-phased with LD information only on haplotypes obtained with genotyping data, with both LD and familial information and on a much larger population. This strategy results in very few mismatches with the phase obtained by Mendelian segregation rules. Finally, we propose a strategy to further improve phasing accuracy based on haplotype clusters obtained with genotyping data.

**Electronic supplementary material:**

The online version of this article (doi:10.1186/s12711-017-0321-6) contains supplementary material, which is available to authorized users.

## Background

Most genotyping technologies provide, for each marker, the combination of marker alleles that are carried by an individual. Haplotype reconstruction for such genotyping data, or phasing, refers to statistical methods that determine which marker alleles were inherited from the same parent and are located on the same homolog. It is an essential step in many applications, including imputation [1], pre-phasing of reference panels [2], estimation of identity-by-descent (IBD) probability for genetic or QTL mapping [3], association analysis (e.g. [4–6]), genomic selection [7–10], studies of genetic diversity, detection of signatures of selection [11, 12] or the study of the recombination process (e.g. [13]).

Most haplotyping methods rely either on familial information (e.g., [14, 15]), linkage disequilibrium (LD, e.g. [16–19]) or both (e.g., [20]). Methods that rely on heuristics (possibly in combination with familial information) have also proven efficient [21–23]. The use of familial information is particularly important to perform haplotype reconstruction at long distances and is extremely precise with large half-sib families whereas LD-based methods are very effective at short distances. Note that the so-called long-range phasing (LRP) methods achieve haplotype reconstruction at long distances without requiring explicit familial information.

In many populations, including livestock species, whole-genome sequencing (WGS) is applied only to a relatively small sample of individuals, because associated costs remain high. In many cases, unrelated reference individuals are selected to capture as much variation from the population as possible [24]. Therefore, the use of familial information might be of little benefit. Consequently, these datasets are most often phased with LD-based methods only (e.g. [25–27]). The small size of the reference population also impacts the efficiency of these LD-based methods and the inferred haplotypes. Improving the phasing accuracy should positively impact all related applications mentioned above. Large samples from a population, including many relatives of these reference individuals, are genotyped with single nucleotide polymorphism (SNP) arrays. As a result, the quality of haplotype reconstruction with such SNP array data is high due to the use of the available familial information. In addition, more genotyped individuals are available to estimate LD patterns (between SNPs on the array).

The main objective of our study was to determine whether phasing of WGS data of reference individuals using their haplotypes that are obtained with genotyping array data as template (hereafter called “scaffold” as in [1, 28]) is more accurate or not. In addition, we evaluated whether phasing based on LD and familial information has an impact on imputation accuracy. Finally, we suggest several possible improvements of the phasing method.

## Methods

### Data

#### Selection of SNPs from WGS data

In the current study, we selected 67 bulls and 24 cows that originated from New-Zealand and were all both genotyped and sequenced at high coverage (15*x* or more) from a larger WGS dataset that was previously used in [29]. It should be noted that, in this study, our aim was to assess the phasing accuracy for WGS genotypes called with relatively high confidence and not for low-fold WGS data. Detailed procedures to generate the WGS data, including DNA extraction, sequencing procedure, alignment, quality score recalibration and variant calling were previously described in [29].

This WGS dataset is composed of 36 Holstein–Friesian (six cows and 30 bulls), 24 Jersey bulls and 31 Holstein–Friesian/Jersey crossbred (18 cows and 13 bulls) individuals. Among these 91 animals, 38 parent-offspring pairs were available for which data was available in the WGS dataset for the sire of 30 animals, for the dam of two animals and for both sire and dam of three animals. These parent-offspring relationships span over several generations (up to four generations) and were used to phase offspring with high confidence based on the Mendelian segregation rules.

When evaluating phasing accuracy on real WGS data, the estimated phasing errors do not result only from genuine phasing errors but also from other sources (e.g. assembly or genotype calling errors), which can blur the genuine phasing errors. To reduce as much as possible, the noise due to other sources of errors, we performed a very stringent data filtering to select the so-called trusted variants (high-confidence variants). For the sake of generality, we also performed a more traditional variant filtering for ease of comparison with other studies and to evaluate phasing in more realistic conditions. In this paper, the WGS dataset always refers to the trusted set of variants, unless explicitly specified.

The stringent filtering rules applied to the 22,228,949 SNPs from the original VCF file are described hereafter. In addition to calibration score, we used VCFtools [30] to select bi-allelic SNPs that:

- are present in other available bovine WGS datasets (the 1000 bull genomes [31] run 2, the Belgian Blue cattle and New-Zealand populations used in [29] and a Dutch Holstein pedigree of 415 individuals reported in [32]);
- are present in the datasets of all 91 individuals used here;
- have a MAF higher than 0.01 (i.e. any SNP for which the minor allele occurred only once was discarded);
- did not deviate from Hardy–Weinberg equilibrium (*p* > 0.05).

In this selection, we retained SNPs that displayed correct Mendelian segregation in the WGS Dutch Holstein pedigree based on the following rules: no parent-offspring incompatibilities (e.g., opposite homozygotes), no deviation from Hardy–Weinberg proportions (*p* > 0.05) and no deviation from expected genotypic proportions in offspring of heterozygous parents (*p* > 0.05). In addition, we excluded those markers associated with a low power to detect possible parent-offspring inconsistencies. Application of these filtering steps reduced substantially the number of SNPs but also the level of genotyping errors.

In addition to variant quality, we also removed some genomic regions that may be incorrectly mapped (errors in the genome assembly). Additional errors were detected based on the following evidences: multiple long runs of homozygosity (ROH) that had been detected with the genotyping array data were heterozygous for some segments of the WGS data, excess of double cross-overs in the WGS Dutch Holstein pedigree compared to the array-based haplotypes, and split reads or unexpected distances between mate-pairs in a WGS mate-pair library.

Finally, SNPs that were retained in the genotyping array dataset (see below) but discarded based on the filtering step mentioned just above were re-introduced in the WGS dataset. Application of the complete series of filtering steps resulted in a final list of 5,185,663 SNPs (thereafter referred to as the trusted set of WGS SNPs) that are listed in Additional file 1: Table S1, whereas, application of only the more traditional filtering steps, i.e. SNPs that were bi-allelic, present in the datasets of all 91 animals, showed no deviation from Hardy–Weinberg equilibrium with *p* > 0.05, and had a MAF higher than 0.01 resulted in 13,175,535 SNPs. The latter set was used only for illustrative purposes (comparisons to other studies) and will be referred to as the traditionally filtered WGS data (see Additional file 2: Table S2).

#### Selection of SNPs from genotyping array

A total of 58,369 animals from Livestock Improvement Corporation (LIC, New Zealand), including the 91 sequenced animals, were genotyped using either the BovineSNP50k (v1 and v2) or the BovineHD genotyping array from Illumina. Only SNPs that were common to the three arrays were retained. We removed SNPs that had a call-rate less than 95%, generated more than 10 Mendelian inconsistencies, were monomorphic or strongly deviated from Hardy–Weinberg equilibrium. In addition, map errors were detected and discarded using LINKPHASE3 [33]. Application of these filters resulted in 37,740 autosomal SNPs. Furthermore, 2455 SNPs that showed more than 4% mismatches between the genotype and WGS data for the 91 individuals were discarded. This final panel of 35,285 phased SNPs will be referred to as genotyping data. As stated above, all the SNPs used in the genotyping data were present in the WGS data.

### Phasing methods applied to genotype and WGS data

#### Phasing of the genotype data using only LD information (GEN-P1)

A first phasing was done for all 58,369 genotyped animals from LIC using SHAPEIT2 [34, 35] and default parameters except for the window size (set to 5 Mb). The originality of this method consists in the possibility to efficiently explore the space of the haplotypes that are consistent with a given genotype. This phasing method is referred to as “GEN-P1” and the results were used as the pre-phase for imputation of the WGS data from the genotyping data using only LD information.

#### Phasing of the genotype data using both LD and familial information (GEN-P2)

As mentioned above, LINKPHASE3 was used to detect and discard map errors. However, the original purpose of this method is to partially phase the genotypes using Mendelian segregation rules and linkage in half-sibs families. After applying this method to the population of 58,369 animals, further haplotype reconstruction was performed by integrating LD information using DAGPHASE [20] and Beagle [16]. The resulting haplotypes were therefore inferred with both familial and LD information to the 35,285 SNPs (missing genotypes being imputed by Beagle). This phasing method is referred to as “GEN-P2” and was used as scaffold for phasing the WGS data panel using both LD and familial information.

#### Phasing of the WGS data using only LD information (WGS-P1)

As for the genotype data, we ran directly SHAPEIT2 on the WGS data. Since the population of WGS animals is relatively small (91 animals), we set the number of conditioning states to the maximum value (182 different haplotypes). The window size was set to 0.5 Mb, as suggested in the SHAPEIT2 documentation for use with sequence data. This phasing is referred to as “WGS-P1” and will also serve as a pre-phasing step for two purposes: (1) for phasing the WGS data using both LD and familial information and (2) as reference for imputation to WGS level using only LD information.

#### Phasing of the WGS data using both LD and familial information (WGS-P2)

This phasing is also achieved using SHAPEIT2 by using the option “call” instead of “phase”. The original aim of this option is to improve genotype calling from low coverage WGS data [28] by applying a technique (haplotype scaffold) that uses the phase of a SNP genotyping array as scaffold. The principle is that the scaffold constraints the space of consistent haplotypes. Each non-overlapping successive segment (at least three sequence SNPs) is then aligned to the scaffold.

In our implementation, the WGS data had a high coverage (≥15*x*), thus the genotypes are coded as integers rather than real dosages and the space of the haplotypes that is consistent with a given genotype (and also with the scaffold) is expected to be much smaller than in the case of low-coverage data.

In our specific case, the advantage of this technique was that it aligned the pre-phased WGS data (WGS-P1) to the GEN-P2 phase (i.e. the scaffold). In the latter, LD information was obtained from many more samples than for WGS-P1 (58,369 vs. 91 for WGS-P1, see Table 1 for more details) but above all, GEN-P2 phase was also based on very accurate familial information and thus, it is expected to be correct at a longer range than GEN-P1 phase. This argument was verified beforehand: close to 50% of the SNPs of the genotyping data had a GEN-P1 phase that was in opposite phase to that obtained based on Mendelian segregation rules. Since GEN-P2 phase was based on pedigree information, obviously it was in complete concordance with the phase that was obtained based on Mendelian segregation rules. Thus, it is recommended to use the GEN-P2 as a scaffold to phase the WGS data.

**Table 1:** Importance of the familial information for the 91 animals of the WGS dataset

	Phased with 58,369 genotyped animals	Phased with 91 sequenced animals
Both parents genotyped	23	3
Only one parent genotyped	67	32
At least one offspring genotyped (average number of offspring^a^)	80 (178.6)	17 (2.2)

^a^The average number of offspring genotyped is the average number of offspring considering only animals with at least one offspring

In our study, all SNPs of the genotype data are included in the WGS data, which results in the WGS-P1 segments (defined as consecutive WGS SNPs for which the closest genotyped SNP is the same) that align on the GEN-P2 phase to be at least 1 bp long. On average, these WGS-P1 segments contain ~140 WGS SNPs and their median length is ~53 kb (see more details in Table 2).

**Table 2:** Size distribution of the WGS segments^a^ encompassed by the scaffold (GEN-P2) (number of SNPs, physical length)

	GEN-P2 scaffold	
Number of WGS SNPs per segment	Minimum	1
	Average	146.97
	Median	110
	Maximum	2241
Singleton segments^b^	Number	145
	Scaffold proportion (%)	0.41
Physical length of segments in bp	Minimum	1
	Average	67,834.76
	Median	53,507
	Maximum	1,703,836
Physical length of non-singleton segments in bp^b^	Minimum	39
	Average	68,114.66
	Median	53,715

^a^WGS segments being defined as all consecutive WGS SNPs of the trusted set of SNPs for which the closest genotyped SNP is the same

^b^“Singleton segments” refers to segments that contain only one SNP from the scaffold, therefore a scaffold SNP encompassing only itself in the WGS data

Regarding the initial phasing of the WGS data, the number of conditioning states was set to 182 and the window size to 0.5 Mb. The number of Markov chain Monte Carlo (MCMC) iterations was optimized using a subset of parent-offspring pairs and subsequently set to 12 burn-in iterations and 30 main iterations (on which haplotypes are averaged). Twelve pruning stages of four iterations each were used for a more parsimonious haplotype graph, as suggested in the SHAPEIT2 documentation.

**Fig. 1:**
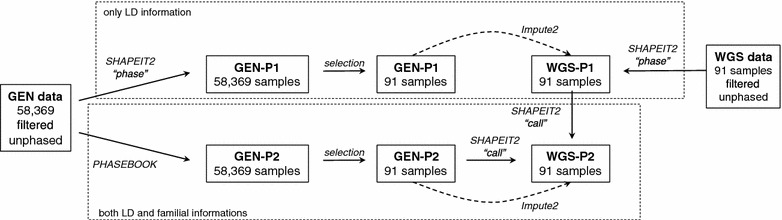
Flowchart of all phasing and imputation steps. Synoptic view of the two phasing strategies (*P1* with LD information only, *P2* with both LD and familial information) applied to the two datasets (*GEN* 50k dense genotype array data, *WGS* whole-genome sequence data) and the two imputation scenarios

Figure 1 provides an overview of all the phasing steps described.

### Assessing phasing accuracy of haplotypes WGS-P1 and WGS-P2

Two criteria were computed to assess and compare the accuracy of the WGS-P1 and WGS-P2 phases: proportion of phasing errors and number of switches. To compute these statistics, the WGS data was divided as follows: of the 91 animals included here, 77 were retained in the training set and 14 were removed because they were parents (10 sires and 4 dams) of 30 animals of the 77 training animals (28 with only one parent known and two with both parents known). Phases WGS-P1 and WGS-P2 were then estimated by using the training set only.

For each of the 30 animals with at least one parent known, WGS-P1 and WGS-P2 were subsequently compared with the phase that was based on the Mendelian segregation rules, which correctly phase the SNPs that are heterozygous in the offspring and homozygous in at least one parent. Such SNPs are referred to as “phasable” and across these 30 animals, they represent on average ~15% of the WGS SNPs for the animals with only one sequenced parent and ~26% for those with two sequenced parents.

The proportion of phasing errors is the proportion of mismatches between the Mendelian phasing and the method under evaluation for the phasable SNPs. Each sequence of one or more consecutive mismatches delimits an incorrect segment, regardless of the distance between the SNPs in that sequence. Conversely, each sequence of one or more consecutive matches delimits a correct segment. The number of switches was recorded per animal as the number of times the phase switches from correct to incorrect or conversely. Segment length distribution was also recorded, as well as the number of singleton segments (i.e. segments containing only one phasable SNP).

### Assessing the impact of the pre-phasing strategy on accuracy of imputation from genotype data to WGS data

To assess whether a pre-phasing strategy based on both LD and familial information improves or not the accuracy of imputation, we compared two scenarios (see Fig. 1):

1. WGS-I1, imputation using WGS-P1 pre-phased haplotypes, i.e. imputation is performed from GEN-P1 to WGS-P1;
2. WGS-I2, imputation using WGS-P2 pre-phased haplotypes, i.e. imputation is performed from GEN-P2 to WGS-P2.

To evaluate the impact of the pre-phasing strategy on imputation accuracy, a 13-fold cross-validation was performed. The imputation to seven target animals from 84 reference animals was repeated 13 times. Pools of seven animals were randomly chosen without repetition, which resulted finally in 91 imputed animals. Imputation was achieved for all 29 bovine autosomes by using Impute2 [1], with an effective population size set to 200, a number of reference haplotypes set to 168, i.e. twice the number of reference animals, and by applying the option “–allow-large-regions” to impute the entire chromosome at once. For each animal, the result is imputed dosage of both phases.

The following statistics were then obtained for all WGS SNPs by comparing the imputed dosages and observed genotypes of the 91 animals: imputation accuracy *r* ^2^, as the squared correlation between imputed dosages and observed genotypes of any WGS SNP, and imputation error rate, as the sum of the residues between imputed dosages and observed genotypes per number of imputed SNP alleles (i.e. twice the number of SNPs).

## Results

### Phasing accuracy

Proportions of phasing errors, numbers of switches and distributions of length of segments are in Tables 3 and 4 for the trusted set of variants. The results indicate that phasing with LD information only (WGS-P1 phase) leads to random assignment of parental origin: about 50% of SNPs are not correctly phased. Conversely, aligning the WGS-P1 phase on the GEN-P2 phase (relying on familial information), i.e. the WGS-P2 phase, results in accurate inference of the parental origin along each chromosome: 99.62% of the phasable SNPs for the animals in the training set are correctly assigned.

**Table 3:** Statistics of phasing results for the two phasing strategies

	Trusted set of variants				Traditional SNP filtering			
	WGS-P1		WGS-P2		WGS-P1		WGS-P2	
	Average	Median	Average	Median	Average	Median	Average	Median
*Proportion of phasing errors*								
Per animal	50.95%	50.13%	0.38%	0.32%	50.80%	50.41%	1.10%	1.04%
*Number of switches*								
Per animal	739.2	631.5	704.5	574	4521	4291	4387.7	4079
Per animal and chromosome	25.49	18.5	24.29	16.5	155.9	105.5	151.3	112

*WGS-P1* phased with LD information only, *WGS-P2* phased with both LD and familial information

**Table 4:** Lengths of segments without switches of the trusted set of WGS SNPs for the two phasing strategies^a^, whether correctly or wrongly phased or both (all)

	WGS-P1			WGS-P2		
	All	Correct	Wrong	All	Correct	Wrong
Original segments						
Physical length^b^						
Avg	3.01	2.96	3.07	3.19	6.11	84.99 kb
Med	4.58 kb	4.75 kb	4.33 kb	3.38 kb	1.74	1 bp
Max	150.73	150.73	123.75	116.39	116.39	42.76
Proportion of singletons^c^	37.52%	37.53%	37.51%	38.82%	12.18%	67.24%
Number of phasable SNPs per segment						
Avg	1048.45	1041.75	1055.17	1098.05	2119.04	8.39
Med	4	4	4	3	644	1
Max	55,553	55,553	50,217	51,340	51,340	835
Number of SNPs per segment						
Avg	6232.95	6134.89	6331.09	6612.58	12,639.78	179.99
Med	12	13	12	8.5	3641	1
Max	308,884	308,884	240,332	231,051	231,051	85,165
After discarding singletons^c^						
Physical length^b^						
Avg	9.48	9.28	9.67	11.02	19.46	0.36
Med	1.25	1.39	1.12	0.26	6.39	0.01
Max	154.47	154.47	147.3	147.3	147.3	42.76
Number of phasable SNPs per segment						
Avg	3185.72	3163.65	3207.84	3652.87	6524.06	28.54
Med	367	418	314	59	2352	4
Max	67,776	67,776	60,281	67,776	67,776	1452
Number of SNPs per segment						
Avg	19,586.14	19,210.75	19,962.22	22,778.53	40,230.9	748.21
Med	2555	2925.5	2306	523	13,713	15
Max	318,618	318,618	304,742	304,742	304,742	85,165
After discarding segments with less than five phasable SNPs and shorter than 5 kb						
Physical length^b^						
Avg	14.78	14.46	15.11	19.36	31.64	0.72
Med	4.62	4.72	4.51	2.28	19.59	0.06
Max	158.12	158.1	158.12	158.24	158.24	42.76
Number of phasable SNPs per segment						
Avg	4956.66	4911.73	5001.86	6400.99	10,581.41	53.68
Med	1489	1584	1401	522	6580	13
Max	73,348	73,348	60,625	67,776	67,776	1452
Number of SNPs per segment						
Avg	30,549.61	29,916.85	31,186.28	40,033.57	65,412.51	1499.57
Med	9884	10,364	9497.5	4913	41,646	138
Max	327,738	327,706	327,738	327,914	327,914	85,165

A segment is defined as a run of consecutive phasable SNPs without switches

^a^ *WGS-P1* phased with LD information only, *WGS-P2* phased with both LD and familial information

^b^Unless specified, all length units are in Mb

^c^“Singleton” refers to segments that contain only one SNP

We also observed differences between WGS-P1 and WGS-P2 in terms of number of switches and lengths of segments but they were not as important as those for phasing errors. The WGS-P2 phase presents fewer switches than the WGS-P1 phase, i.e. ~1.2 switches less per chromosome. On average, the distances between consecutive switches are larger for the WGS-P2 phase (3.19 Mb) than for the WGS-P1 phase (3.01 Mb.) We also found that any WGS SNP was located, on average, at 7.8 Mb of the closest switch for the WGS-P2 phase whereas it was only at 6.7 Mb for the WGS-P1 phase (Table 5). In the next section, we assess whether this had an influence on imputation.

**Table 5:** Distance^a^ between any SNP of the trusted set of WGS SNPs and the closest switch

	WGS-P1	WGS-P2
Average	6.74	7.77
Median	3.49	4.08
Maximum	150.81	98.69

Distances are estimated on 30 animals of the training population

^a^In Mb

**Fig. 2:**
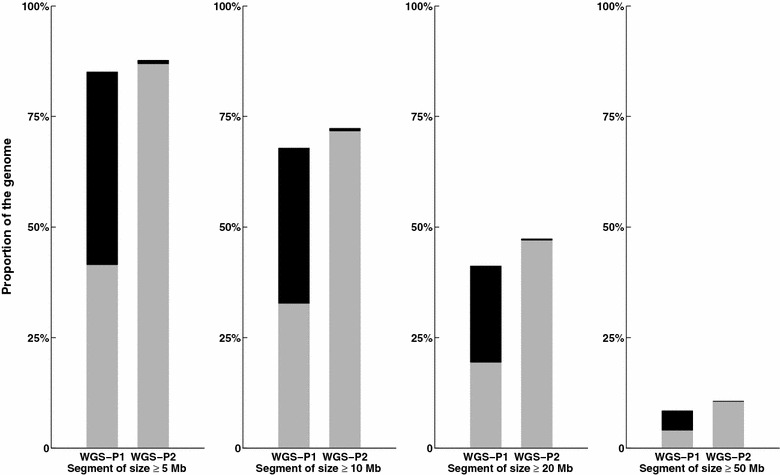
Proportion of the genome by class of size of phased segments. Proportions of the genome in segments that are longer or equal to 5, 10, 20 or 50 Mb, regardless of whether they are correctly (*grey*) or incorrectly (*black*) phased, when phasing the WGS data using only LD information (WGS-P1) or both LD and familial information (WGS-P2)

Figure 2 shows the proportions of segments that are longer than 1, 5, 10 or 20 Mb with both WGS-P1 and WGS-P2, and whether these are correctly or incorrectly phased. Segment lengths that were equal to or longer than 50 Mb (~15% of the genome) were all correctly phased in the case of WGS-P2.

However, the median and maximum segment lengths were greater for the WGS-P1 phase, which is illustrated by the proportion of singleton segments, i.e. they are slightly more numerous for WGS-P2 than for WGS-P1 (respectively, 37.1 and 35.7% of the total number of segments), but about 84% of the singleton segments obtained with WGS-P2 are incorrectly phased. The difference in average segment length between WGS-P1 and WGS-P2 is larger if we consider that only segments below a threshold length are phasing errors: 9.5 versus 11 Mb when discarding singletons and 15 versus 19 Mb when discarding singletons and small segments (maximum 5 phasable SNPs and 5 kb). Discarding singletons and small segments leads to an average length of almost 32 Mb for WGS-P2 segments that were correctly phased.

The results obtained with more traditional variant filtering rules (thus, containing more noise due to errors that do not depend on the phasing method) are in Tables 3 and 6. Compared to the results obtained with the trusted set of variants, we observed more errors. First, correct identification of parental origin drops slightly i.e. to 98.9% of the variants with WGS-P2, whereas parental origin remains randomly assigned with WGS-P1 (Table 3). The proportion of phasing errors increases by a ratio close to the number of SNPs (on average, 2.89 more phasing errors with 2.54 more SNPs). The increase in number of switches (per animal or per animal and per chromosome) is much more pronounced: on average 7.26 more switches than with the stringent set of SNPs (Table 3). When relaxing filters for SNP selection, the size of the segments (whether correctly phased or not) drops substantially from ~3 to ~0.5 Mb for both phases WGS-P1 and WGS-P2, although less in terms of number of SNPs (on average, from 2.38 to 2.85 less SNPs, for phases WGS-P1 and WGS-P2 and for number of all SNPs or only phasable SNPs, see Table 6). These reductions in overall performances indicate that, with such traditional filtering, many errors remain in the dataset (probably due to errors in the assembly or in the genotype calling), which makes the comparison of methods more difficult. Still, the strategy that relies on familial information results in more accurate phasing: WGS-P1 tends to produce slightly shorter segments than WGS-P2. As for the trusted set of variants, segments with a correctly assigned parental origin are on average about two times longer when phasing relies on familial information (WGS-P2).

**Table 6:** Lengths of segments without switches obtained with WGS SNPs selected with more traditional filtering rules for the two phasing strategies^a^, whether correctly or wrongly phased or both (all)

			WGS-P1			WGS-P2		
			All	Correct	Wrong	All	Correct	Wrong
Original segments								
Physical length^b^								
Avg			0.50	0.49	0.50	0.51	0.99	0.04
Med			346 bp	297 bp	404 bp	396 bp	223.51 kb	1 bp
Max			34.88	34.88	21.33	25.64	25.64	14.65
Proportion of singletons^c^			40.76%	41.05%	40.48%	40.61%	16.11%	65.31%
Number of phasable SNPs per segment								
Avg			373.70	370.72	376.69	384.98	758.49	8.40
Med			2	2	2	2	131	1
Max			29,712	29,712	26,646	32,839	32,839	1813
Number of SNPs per segment								
Avg			2618.11	2593.29	2642.94	2704.68	5179.74	209.22
Med			4	4	4	4	1228.5	1
Max			158,630	158,630	113,469	134,130	134,130	84,929
After discarding singletons^c^								
Physical length^b^								
Avg			1.88	1.87	1.9	1.94	3.64	0.19
Med			0.05	0.05	0.05	0.03	1.04	1.92 kb
Max			63.16	55.98	63.16	85.72	85.72	31.87
Number of phasable SNPs per segment								
Avg			1335.70	1325.27	1346.13	1373.47	2675.79	28.52
Med			14	14	14	10	659	3
Max			74,067	57,000	74,067	97,334	97,334	2787
Number of SNPs per segment								
Avg			9895.56	9823.59	9967.51	10,208.87	19,121.57	1004.46
Med			255	241	266	153	5657	13
Max			325,812	293,893	325,812	437,121	437,121	141,991
After discarding segments with less than five phasable SNPs and shorter than 5 kb								
Physical length^b^								
Avg			4.34	4.31	4.36	4.84	8.84	0.50
Med			0.95	0.95	0.95	0.44	3.52	494.45 kb
Max			121.41	121.41	116.11	121.41	121.41	33.07
Number of phasable SNPs per segment								
Avg			3045.22	3024.33	3066.06	3388.28	6444.96	69.46
Med			318	310.5	325	78	2311	10
Max			110,344	110,344	86,208	112,056	112,056	2793
Number of SNPs per segment								
Avg			22,773.56	22,633.66	22,913.14	25,438.65	46,480.17	2592.66
Med			4951	4939	4971	2206	19,046	254
Max			605,700	605,700	601,045	653,272	653,272	160,715

A segment is defined as a run of consecutive phasable SNPs without switches

^a^WGS-P1: phased with LD information only; WGS-P2: phased with both LD and familial information

^b^Unless specified, all length units are in Mb

^c^“Singleton” refers to segments that contain only one SNP

### Accuracy of imputation of the WGS data

Imputation accuracies (measured as *r* ^2^) are in Table 7 for each imputation scenario WGS-I1 (only LD information) and WGS-I2 (both LD and familial information), on both sets of SNPs. Although the scenario that indirectly accounts for familial information (through the use of a scaffold that exploits both familial and LD information) performs better than the other scenario, this difference is small with the trusted set of SNPs: 90.65 for WGS-I2 versus 90.47% for WGS-I1. The overall imputation error rate is 1.70% for WGS-I1 and 1.67% for WGS-I2, averaged per chromosome and animal, i.e. the scenario that indirectly accounts for familial information reduces error rate by ~2% (in relative terms).

**Table 7:** Imputation reliability (measured as *r* ^2^ and given in %) for the two scenarios^a^ of imputation

	Trusted set of variants						Traditional variant filtering					
	N	WGS-I1^a^		WGS-I2^a^		D_I2-I1_^b^	N	WGS-I1^a^		WGS-I2^a^		D_I2-I1b_
		Avg *r* ^2^	Med *r* ^2^	Avg *r* ^2^	Med *r* ^2^			Avg *r* ^2^	Med *r* ^2^	Avg *r* ^2^	Med *r* ^2^	
Overall	5,149,267	90.47	93.63	90.65	93.81	0.18	13,129,937	88.66	93.57	89.07	93.87	0.41
NMA = 2^c^	79,755	56.74	67.47	59.98	86.76	3.24	680,303	63.04	80.94	67.95	96.96	4.91
NMA = 3^c^	78,933	69.10	71.02	70.78	75.35	1.68	510,080	77.28	92.25	79.00	95.49	1.72
0.01 < MAF ≤ 0.05	644,224	77.57	85.82	78.45	87.11	0.88	3,278,384	79.56	91.35	81.01	94.27	1.46
0.05 < MAF ≤ 0.10	673,955	89.15	91.65	89.21	91.81	0.06	2,047,206	89.91	92.82	90.07	93.15	0.16
0.10 < MAF	3,831,088	92.87	94.20	92.95	94.30	0.08	7,804,347	92.15	93.91	92.19	93.96	0.04
First Mb	48,089	85.40	90.90	87.56	92.84	2.15	134,266	83.17	90.10	85.19	92.27	2.01
Last Mb	53,502	87.94	91.55	88.45	92.05	0.51	155,246	85.85	91.26	86.53	91.88	0.68
Between first and last Mb	5,045,959	90.55	93.68	90.70	93.84	0.16	12,840,425	88.75	93.62	89.14	93.91	0.39

^a^WGS-I1: imputation from GEN-P1 to WGS-P1 (using only LD information); WGS-I2: imputation from GEN-P2 to WGS-P2 (using both LD and familial information)

^b^D_I2-I1_: difference of average *r* ^2^

^c^NMA: number of occurrences of the minor allele

However, the difference in imputation accuracy between scenarios is larger for specific classes of SNPs, for which both imputation *r* ^2^ are lower. WGS-I2 results in an imputation accuracy (measured as *r* ^2^) that is 0.88% higher than that for WGS-I1 for variants with a MAF between 1 and 5%. This difference is mainly due to the rarest SNPs being retained in the WGS dataset (two occurrences of the minor allele). For this particular class of SNPs, the median values of *r* ^2^ are 67.47 and 86.78% for WGS-I1 and WGS-I2, respectively.

Both ends of all chromosomes also present lower *r* ^2^ and larger differences between methods: on average, *r* ^2^ is 2.15 and 0.51% higher for the first and last Mb of each chromosome, respectively, with WGS-I2.

When relaxing filters for the selection of WGS SNPs (more traditional variant filtering rules), the average imputation accuracy (measured as *r* ^2^) drops slightly for both imputation scenarios, but the difference between scenarios is more important than when a more stringent selection is applied: 89.07 for WGS-I2 versus 88.66% for WGS-I1. This difference is probably due to the classes of rare variants, since they are much more frequent in this dataset (e.g. ~8.5 more SNPs of the rarest class).

## Discussion

### Integrating familial information in phasing of WGS data results in accurate haplotypes with sparse phasing errors

The main idea of our new strategy is to indirectly use familial and LD information from genotyped populations to improve phasing of a smaller population of whole-genome sequenced reference individuals in a two-step procedure. It should be noted that the strategy is not restricted to WGS data; for instance, phasing of a HD panel can be improved with information from a larger 50k panel. The reasoning is that genotyped populations are larger and thus more familial information (more genotyped parents and more genotyped offspring) is available. For instance, 80 of the 91 animals used in this study have offspring in the genotyped population (178.6 offspring on average, see Table 1). Within the population of animals with sequence data, this number would drop to 17 animals (with 2.2 offspring on average). Moreover, with a larger population, there are more records and a larger variety of haplotypes represented to infer the LD structure. Therefore, haplotype reconstruction in these larger genotyped populations has proven particularly efficient in pedigreed populations and such haplotypes would be good scaffolds (or anchors) to phase the WGS data. Another key point is that we assume that LD-based methods are able to correctly phase segments that are a few Mb long (but not at long range). As long as these correctly phased segments each contain a few SNPs from the lower-density panel, it should be possible to infer their parental origin based on the phased genotype data. Our results prove that these hypotheses are valid for a bovine dataset and that our strategy results in WGS haplotypes being correctly phased along the entire chromosome except for a few small segments (most often singletons).

First, we determined the range of correct phasing with LD-based methods on WGS data. Generally, methods that are designed for phasing and imputation in human datasets (e.g. [2, 16, 17]) are used in livestock populations without knowing this information. Here we show, that LD-based methods work quite well, resulting in 3-Mb long correctly phased segments. This average value includes singletons and other very small errors and more than 80% of the genome lies in segments that are larger than 5 Mb (see Fig. 2).

**Fig. 3:**
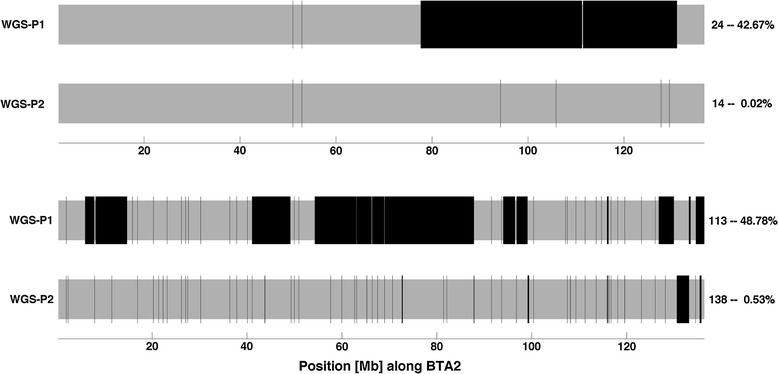
WGS-P1 and WGS-P2 phases of bovine autosome 2 for two animals of the training set. Consecutive SNPs with phase in compliance with Mendelian segregation rules delimit correct segments (*in grey*); conversely, consecutive markers with phase not in compliance with Mendelian segregation rules delimit incorrect segments (*in black*). Corresponding number of switches and proportion of errors are indicated on the *right side* of each phase

Next, we showed that our strategy improves phasing accuracy: a larger fraction of the genome lies within long correctly phased fragments (more than 75% of the genome lies within segments that are longer than 10 Mb) and fewer switches are observed. However, the main benefit is that the parental origin is correctly inferred across the entire chromosome. The phasing errors are mostly associated to small segments: ~70% of these incorrect segments are singletons and ~81% contain five or less phasable SNPs. An illustration of these results for two animals with different profiles is in Fig. 3, i.e. with our new strategy, the chromosome shown for the first animal is divided into 15 segments (14 switches) and all segments that are assigned an incorrect parental origin contain three or less phasable SNPs (only one for most of them), whereas for the second animal there are many more switches (138) but the size of incorrectly phased segments remains small in general.

We suggest that all LD-based phasing methods should offer the possibility to incorporate external phasing information (such as haplotype information inferred from low-density panels), for instance as scaffold. This is often not possible with software programs that are primarily designed for human genetics studies. Popular rule-based phasing and imputation methods that are commonly used in animal breeding genetics such as FImpute [23], findhap [36] or AlphaPhase [21] use information from relatives genotyped at lower marker density to phase animals in the reference panel. A recent study [37] compared haplotypes that were obtained from genotyping array data with such methods to those obtained with LD-based methods and found that FImpute [23] achieved a more accurate phasing than other methods when at least one parent was genotyped.

Improvement of phasing accuracy should positively impact all applications using haplotypes. For instance, for the detection of signatures of selection, it is important that haplotypes are not subdivided into smaller segments. Larger correctly phased segments allow to better identify IBD relationships and to better cluster local haplotypes for imputation, association studies and genomic selection. These applications would work nicely for SNPs that are located in the center of segments, thus only SNPs that are closer to the segments’ boundaries (closer to switches) would remain problematic. LD-based methods result in many more such switches that might locally impact haplotype-based applications (our results show that our two-step strategy increases the proportion of SNPs that are distantly located from switches). In addition, the presence of singletons (as observed with our new strategy) is often well handled by imputation or haplotype clustering methods that accommodate for genotyping errors.

The major difference between our strategy and LD-based methods is the ability to correctly infer the parental origin along the entire length of chromosomes and we showed that this could be improved from ~50 to ~99.62% of the WGS SNPs of the trusted set of variants. Correct parental origin is important for disease mapping (e.g., if it is known that the causal variant is transmitted through the maternal path), when breed origin of different haplotypes must be determined in multiple-breed crosses (e.g. [38]), when studying parental imprinting (e.g., [39]) or when estimating parent-of-origin effects for allele-specific gene expression [40]. For recent mutations in common haplotypes, parental origin and accurate long-distance haplotyping are also essential to determine whether the original or mutated version of the haplotype was inherited.

In the current study, the method was applied to improve phasing of WGS genotypes that are known with relatively high confidence (coverage ≥ 15*x*) and our conclusions are restricted to this situation. Originally, the method was implemented in SHAPEIT2 to improve genotyping calling with low-fold sequencing data. Scaffolds of haplotypes obtained on larger genotyped populations and with familial information may provide even more benefits when used with low-fold sequencing data. In such a case, the scaffold would be used to improve genotype calling, to impute missing genotypes and to perform haplotype reconstruction in the reference panel. With a view to extend the method to low-coverage SNPs, additional phase information could be provided directly from sequence reads (e.g. [41]).

### Improving haplotype pre-phasing has a marginal impact on imputation accuracy

The imputation accuracy achieved in our study is higher than that reported in other recent studies in cattle [25, 31, 42], both with the trusted set of variants (*r* ^2^ = 0.9065) or with more traditional variant filtering rules (*r* ^2^ = 0.8907). It is worth noting that the above-mentioned studies impute data from high-density SNP panels (777k SNPs). For instance, the ratio between number of imputed and reference SNPs on bovine autosome 29 is equal to 28.5 in [25], 46.2 in [31] and 427.1 in our study (with the trusted set of variants), thus, there are respectively 15.0 or 9.2 times more SNPs imputed from a single SNP in our study. However, comparisons are difficult, since populations and the sizes of the reference populations differ. In addition, results are often expressed as correlations (*r*) whereas we used squared correlations (*r* ^2^).

Surprisingly, improved haplotype pre-phasing had only a marginal impact on imputation accuracy. Imputation relies on shared identical-by-state (IBS) segments between target and reference animals on the low-density panel (here, the genotyping data). Therefore, we computed the average length of the longest IBS segment shared by any target haplotype from the phased genotype data (GEN-P1 or GEN-P2) and one of the 168 reference haplotypes (WGS-P1 or WGS-P2 phases obtained for the trusted set of variants) on all SNPs of the genotype data. The longest IBS segment was on average 43.4 Mb long for the GEN-P2 phase (including familial information) versus 26.3 Mb for the GEN-P1 phase. The good performances of the WGS-I1 scenario (based on GEN-P1 phase) could be due to the fact that the length of IBS segments between any target and the most similar reference haplotype is already sufficiently long (although shorter than with the WGS-I2 scenario).

### Using additional information to improve the scaffold

A possibility to further improve phasing of WGS data and imputation accuracy could be to further enrich the scaffold with SNPs that are phased with high confidence. As an illustration of this perspective, we previously observed that for SNPs on genotyping arrays, the LD between haplotype clusters (hereafter called ancestral haplotypes—AHAP) and underlying variants was high [6]. When there is a perfect match between such AHAP and variants from the WGS (each AHAP being perfectly associated with one SNP allele), we can use these AHAP to determine the parental origin of the corresponding SNP alleles. We determined that 28% of the SNPs from the trusted set presented such a perfect association with a set of 50 AHAP and that using these haplotypes to phase these 28% SNPs resulted in a phasing accuracy of 99.9% (data not shown). Consequently, we considered that these SNPs could be added to the scaffold (resulting in a scaffold of 1,485,758 WGS SNPs of the trusted set). Phasing and imputation accuracy were improved when using this new scaffold. Parental origins were correctly assigned for 99.72% of the SNPs (compared to 99.62% previously), less switches were observed (20.83 vs. 24.29 switches per chromosome) and imputation accuracy increased from 90.65 to 90.91%. This was even more pronounced for rare variants: imputation accuracy for the rarest variants (two occurrences of the minor allele) had a median imputation accuracy *r* ^2^ equal to 94.71% compared to 86.76% with the first scaffold. These results suggest that a strategy that relies on a scaffold of variants phased with high confidence can be further extended to other sources of information as long as they provide accurate phasing information.

## Conclusions

In this paper, we describe a multi-step strategy to take both LD and familial information into account when phasing WGS data. The strategy relies on the use of a 50k genotyping array, phased on a large population (including many relatives of the sequenced individuals) and using both LD and familial information, as haplotype scaffold. This strategy results in a very low proportion of mismatches with the phase obtained by Mendelian segregation rules (0.32% on average). It also results in longer correctly phased segments than a method that relies on LD only. The majority of the errors results from single SNP errors. Imputation with such an improved pre-phasing step was slightly better than with a traditional pre-phasing step. This small difference between the two imputation scenarios may be explained by the fact that even without a scaffold, correctly phased segments are already long enough for accurate imputation. Finally, we propose an additional strategy to further improve both haplotype reconstruction and imputation of WGS data that relies on haplotype clustering based on the 50k genotyping array data.

## Additional files

**Additional file 1: Table S1. **Detailed list of the trusted set of SNPs from WGS data. Full list of the 5,185,663 SNPs referred in the current study as the trusted set of SNPs from WGS data (chromosome, position in base-pairs, identifier, reference and alternative alleles).

**Additional file 2: Table S2. ** Detailed list of the panel of SNPs from WGS data obtained with more traditional filtering rules. Full list of the 13,175,535 SNPs obtained with more traditional variant filtering rules and used in this study for illustrative purpose (chromosome, position in base-pairs, identifier, reference and alternative alleles).
